# Postpartum health-care utilization and blood pressure control by antihypertensive agent in hypertensive disorders of pregnancy

**DOI:** 10.1016/j.ajogmf.2025.101836

**Published:** 2025-11-07

**Authors:** Carrie Bennett, Lara Lemon, Kripa Venkatakrishnan, Sanjana Ghosh, Hyagriv Simhan, Alisse Hauspurg

**Affiliations:** Department of Obstetrics, Gynecology and Reproductive Sciences, Magee-Womens Hospital, University of Pittsburgh Medical Center, Pittsburgh, PA; Department of Obstetrics, Gynecology and Reproductive Sciences, Magee-Womens Hospital, University of Pittsburgh Medical Center, Pittsburgh, PA; Department of Clinical Analytics, University of Pittsburgh Medical Center, Pittsburgh, PA; Department of Clinical Analytics, University of Pittsburgh Medical Center, Pittsburgh, PA; University of Pittsburgh School of Medicine, Pittsburgh, PA; Department of Obstetrics, Gynecology and Reproductive Sciences, Magee-Womens Hospital, University of Pittsburgh Medical Center, Pittsburgh, PA; Department of Obstetrics and Gynecology, Warren Alpert Medical School of Brown University, Providence, RI

**Keywords:** hypertensive disorders of pregnancy, gestational hypertension, pre-eclampsia, chronic hypertension, labetalol, nifedipine, hospital readmission, therapeutic intensity score

## Abstract

**BACKGROUND:**

Hypertensive disorders of pregnancy are a significant contributor to maternal morbidity and mortality in the postpartum period and are the leading cause of postpartum readmission following delivery hospitalization. At present, there remains uncertainty regarding differences in postpartum health care utilization for individuals discharged on specific antihypertensive agents while enrolled in an institutional remote blood pressure (BP) monitoring program.

**OBJECTIVE:**

To evaluate rates of postpartum hospital readmission and emergency room (ER) utilization for individuals with a hypertensive disorder of pregnancy (HDP) enrolled in remote BP monitoring after discharge on labetalol vs nifedipine.

**STUDY DESIGN:**

We performed a retrospective cohort study to evaluate outcomes associated with the type of antihypertensive medication at discharge in postpartum individuals delivering between April 2019 and June 2023 at a single institution. The exposure of interest was discharge on single-agent labetalol vs nifedipine. Individuals were included who were diagnosed with hypertension (HTN) in pregnancy (including pre-pregnancy HTN) and were enrolled in our institution’s remote BP management program. Our institution’s remote program monitors BP up to 6 months postpartum. Individuals were excluded if they were prescribed antihypertensives antenatally. We compared demographics, clinical outcomes, and home BP measures between groups. The primary outcome was postpartum hospital readmission and ER visits within 8 weeks of delivery. Multivariate logistic regression models adjusted for severity of HDP, TIS, race, and BMI.

**RESULTS:**

1507 individuals were included; 488 (32.4%) were discharged on labetalol and 1019 (67.6%) on nifedipine. Those discharged on labetalol had higher BMIs and higher rates of pre-pregnancy HTN. Compared to individuals discharged on labetalol, those discharged on nifedipine were less likely to have a postpartum hospital readmission (9.2% vs 3.9%, *P*<.001). In multivariate logistic regression models, discharge on labetalol was associated with a significant increased odds of postpartum hospital readmission [aOR 2.70 (95% CI 1.57–4.65)] but not ED visits [aOR 1.01 (95% CI 0.67–1.52)] compared with discharge on nifedipine. These findings were similar with stratification by pre-pregnancy HTN. Upon analysis of home postpartum BP data, we found that the proportion of blood pressures in the severe range by each postpartum day was higher in the first ten days postpartum for those discharged on labetalol as compared to nifedipine.

**CONCLUSION:**

Discharge on labetalol was associated with an increased odds of postpartum hospital readmission following a HDP compared with discharge on nifedipine. Our findings suggest this may be driven by more severe hypertension on home monitoring by each day postpartum in the first ten days postpartum.

## Introduction

Hypertensive disorders of pregnancy (HDP) are both prevalent and increasing, affecting approximately 16% of delivery hospitalizations.^1^ HDP are strongly associated with maternal morbidity and mortality, with 32% of maternal deaths carrying a diagnostic code of HDP.^1^ Additionally, HDP in the postpartum period is the leading cause of readmission following delivery hospitalization, comprising greater than 20% of hospitalizations within 30 days.^2^ Wide-scale implementation of institutional home blood pressure monitoring programs in the postpartum period have been shown to yield sizable benefits. Not only have they proven feasible with demonstrated high patient adherence, retention, and satisfaction, but they have also been associated with a decrease in postpartum readmission rates, a higher attendance at postpartum visits, and higher adherence to medication dosing strategies.^3–7^ Further, remote blood pressure monitoring has been shown to be cost-effective given its reduction in health care resource utilization.^8–9^

Recent literature has explored the relationship between specific antihypertensive agent use at discharge and the rate of hospital readmission, noting significantly higher rates of readmission with discharge on oral labetalol as compared with nifedipine.^10–12^ Prior studies have not examined whether these differences persist in individuals participating in remote BP monitoring programs. Given that home BP monitoring is associated with lower readmission rates and improved BP control, it is possible that these findings may not persist in a remotely monitored population due to the ability to titrate antihypertensive medications remotely. Accordingly, we aimed to determine if health care utilization, defined as emergency room visits and postpartum readmission, differed among patients discharged on oral labetalol compared with oral nifedipine XL in the context of a robust remote monitoring program.

## Materials and methods

This is a retrospective cohort study of postpartum individuals diagnosed with hypertension in pregnancy, including pre-pregnancy hypertension, enrolled in our institution’s remote BP monitoring program who were discharged on oral labetalol or oral nifedipine XL at a tertiary, university-affiliated medical center from April 2019 to June 2023.

We analyzed individuals throughout the postpartum course from the time of hospital discharge through 8 weeks postpartum. Individuals with a diagnosis of hypertensive disorder of pregnancy and/or chronic hypertension were included if they were discharged on oral labetalol or nifedipine and were concurrently enrolled in our institution’s remote home blood pressure monitoring (HBPM) program, which has been previously described.^3–4^ Individuals were followed for 8 weeks to maximize inclusion of the primary outcome. Exclusion criteria included hospital discharge on multi-agent oral antihypertensive therapy, discharge on oral antihypertensive agents other than labetalol or nifedipine, or use of an oral antihypertensive agent in the antenatal period. Individuals discharged on multi-agent antihypertensive therapy were excluded to isolate the effects of labetalol or nifedipine monotherapy.

An individual was diagnosed with a hypertensive disorder of pregnancy or chronic hypertension per standard criteria as defined by the American College of Obstetricians and Gynecologists (ACOG). Accordingly, a hypertensive disorder of pregnancy was defined as new-onset hypertension after 20 weeks of gestation.^13^ Chronic hypertension was defined as hypertension diagnosed before pregnancy or before 20 weeks of gestation.^14^

Briefly, to be included in our remote monitoring program, individuals must speak English, Spanish, or Portuguese and have access to a text messaging-enabled mobile device. The unit nurse educators train patients on how to use the BP device prior to discharge. Following discharge, participants are prompted via a secure text message integrated into the electronic medical record to check their BP. Self-measured BPs are sent via text to the medical record; all procedures in place are HIPAA compliant. Our adapted MWH HBPM program involves BP monitoring twice per day for 7 days/week for the first two weeks postpartum and twice per day two days per week the remainder of the first 6 months postpartum. Management of BP is overseen by 4 Maternal-Fetal Medicine physicians using standard BP management algorithms for HDP, which have been previously published.^15^

The primary study outcome was hospital readmission and ER utilization within 8 weeks of delivery. Secondarily, we sought to evaluate the proportion of home blood pressures in the severe range as well as compare therapeutic intensity score (TIS) of the prescribed medications. We repeated our analyses with stratification by pre-pregnancy hypertension. Severe range blood pressures were defined as systolic blood pressure greater than or equal to 160 mmHg and/or diastolic blood pressure greater than or equal to 110 mmHg.^13^ The proportion of severe range blood pressures was calculated using postpartum BP data for each postpartum day and compared by antihypertensive agent at discharge. TIS is a validated measure that is used to quantify the relative amount of medication a patient receives in the context of varying dosing strategies. To calculate TIS, the prescribed total daily dose for the antihypertensive medication (labetalol or nifedipine XL) was set as the numerator and the maximum daily dose for that medication was set as the denominator.^16^ Per the Food and Drug Administration guidelines, the maximum daily dose for labetalol was defined as 2400 milligrams and the maximum daily dose for nifedipine XL was defined as 120 milligrams.^17^ TIS scores were calculated for each individual based upon the dose of antihypertensive medication at discharge and were compared among groups. Initiation of antihypertensives prior to discharge from delivery hospitalization was at provider discretion. There was no standardization of postpartum blood pressure thresholds for antihypertensive initiation nor standardization of initial antihypertensive dosing strategies. Additionally, there was no standard protocol for furosemide initiation postpartum.

In a secondary analysis, we limited our readmissions and ED visits to those that were reviewed and adjudicated to be hypertension-related. An admission was defined as hypertension-related if elevated blood pressure was the presenting reason, if blood pressure was greater than or equal to 140/90, or if a patient received antihypertensive medications.^18^

Maternal, obstetric, and socioeconomic data were obtained from the electronic medical record and, subsequently, the Clinical Data Warehouse at UPMC. The program is approved by the UPMC Quality Improvement Review Committee, and this study was approved by the University of Pittsburgh Institutional Review Board. The data collection and analysis were approved as an exempt study posing no greater than minimal risk. Written informed consent was not required, in accordance with 45 CFR §46.

Between-group comparisons were performed using χ^2^ tests for categorical data, 2-sample *t* tests for continuous normally distributed data, and Wilcoxon rank-sum tests for continuous nonparametric data. We used logistic regression to model odds of ED visits and postpartum readmission within the UPMC Hospital system within the first 8 weeks postpartum. Models were adjusted for predefined covariates known to be associated with antihypertensive type, BP, postpartum ED visits, and readmissions, including maternal pre-pregnancy BMI, self-reported race, hypertensive disorder severity, and therapeutic intensity score. Hypertensive disorder severity was identified through use of intrapartum magnesium sulfate, as these patients were characterized as having preeclampsia with severe features. Self-reported race was considered a surrogate for social and structural determinants of health rather than a biologic indicator. We performed an interaction analysis to evaluate whether the presence of chronic hypertension resulted in effect modification for the association between type of antihypertensive medication prescribed and postpartum ED visit or readmission. All analyses were performed using Stata IC software package version 16 (StataCorp LP).^19^ A p-value less than 0.05 was considered statistically significant.

## Results

In total, 1,507 individuals met inclusion criteria and were included in the final analysis, with 488 (32.4%) individuals discharged on single-agent oral labetalol following hypertensive disorder of pregnancy diagnosis and 1,019 (67.6%) individuals discharged on single-agent nifedipine. There were no temporal trends identified in prescribing patterns of labetalol and nifedipine throughout the study period, with this distribution of labetalol and nifedipine prescriptions remaining similar throughout the study period.

Individuals in both groups were similar in age, parity, gestational age at delivery, insurance status, incidence of gestational diabetes, and mode of delivery (Table 1). Median early pregnancy body mass index (BMI) was higher in those on labetalol (29.5 kg/m^2^ [IQR 24.7–36.8] vs 28 kg/m^2^ [IQR 23.3–33.8], *P*<.001). Additionally, individuals on labetalol were more likely to have pre-pregnancy hypertension (34.0% vs 18.8%, *P*<.001). Individuals discharged on nifedipine were more likely to have received inpatient magnesium sulfate therapy (64.9% vs 58.6%, *P*=.02). Additionally, median therapeutic intensity score was higher for nifedipine than labetalol, respectively (0.25 [IQR 0.25-0.50] vs 0.25 [IQR 0.17-0.33], *P*<.001).

Emergency room utilization within 8 weeks postpartum was similar among groups, with an incidence of 11.5% for those on labetalol and 10.4% for those on nifedipine (*P*=.53; Table 2). Hospital readmission within 8 weeks postpartum occurred in 9.2% of patients on labetalol and 3.9% of patients on nifedipine (*P*<.001; OR 2.49, 95% CI 1.60–3.86). This finding persisted after adjustment for TIS, disease severity, self-reported race, and BMI (*P*<.001; aOR 2.70, 95%CI 1.57–4.65). In total, 88% (75/85) of the readmissions were hypertension-related. When we repeated our primary analyses limited to those with hypertension-related readmission or ER visits, our findings were unchanged (Supplemental Table 1).

Interaction analysis revealed that the relationship between type of medication at discharge and postpartum readmission was similar regardless of the presence of pre-pregnancy hypertension (interaction *P*=.48). As such, the direction of the relationship of postpartum readmission by antihypertensive agent persisted for individuals with chronic hypertension and without chronic hypertension, though was no longer statistically significant in the smaller population of individuals with chronic hypertension (Supplemental Tables 2–3).

**FIGURE 1:**
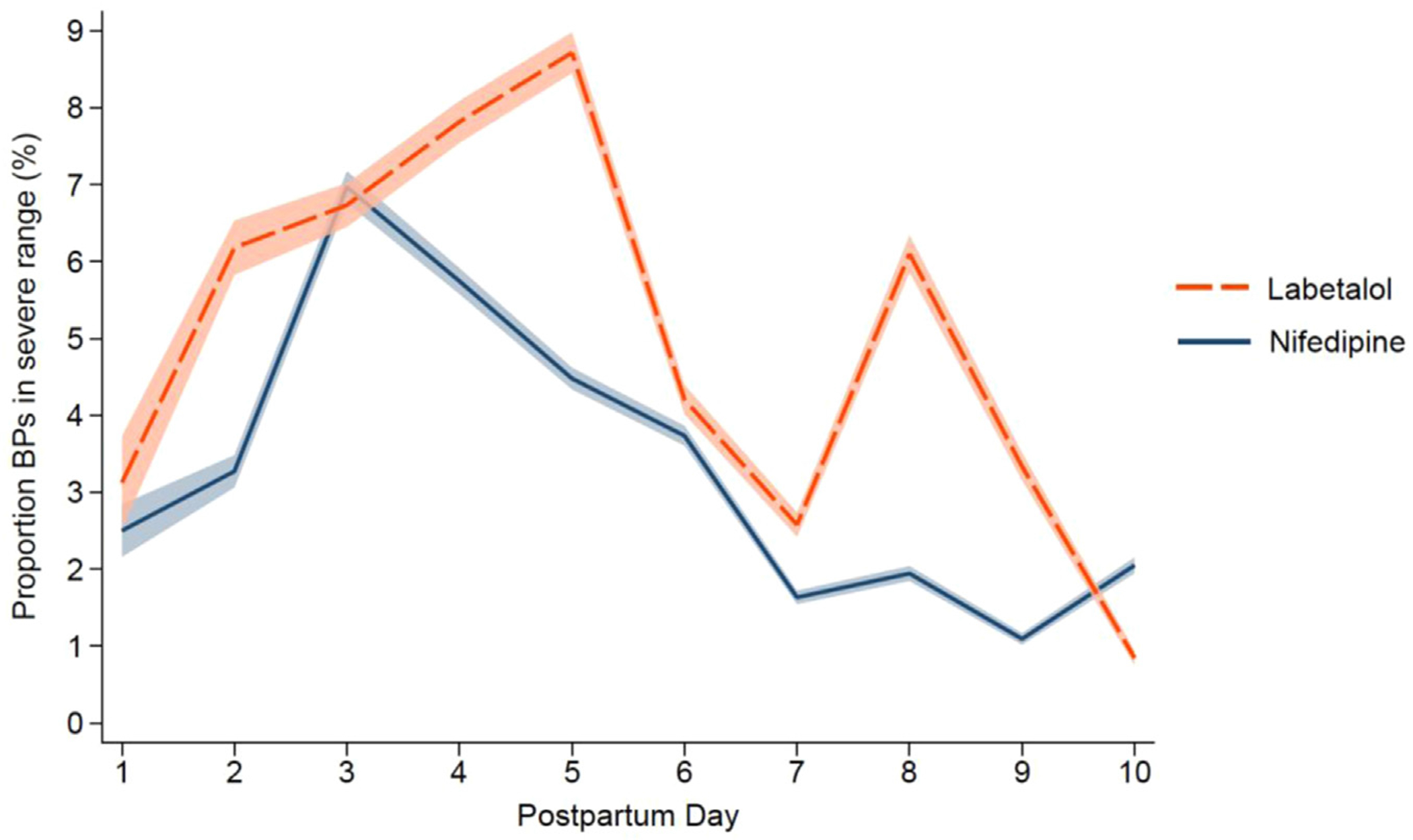
Proportion of blood pressures in the severe range by antihypertensive agent at discharge

Recorded episodes of severe hypertension through outpatient blood pressure monitoring occurred at similar rates among groups (Table 3). Twenty-two percent of patients discharged on labetalol had one or more recorded episodes of severe hypertension captured through inpatient monitoring and/or outpatient remote blood pressure monitoring through the entire monitoring period compared to eighteen percent of individuals discharged on nifedipine (*P*=.07). To explore differences in severe hypertension within the immediate postpartum period, we compared rates of severe hypertension by each postpartum day across medication types. This blood pressure analysis began on postpartum day 1 and included blood pressure values from an individual’s inpatient admission. As expected, the proportion with severe hypertension was highest on postpartum days 3-6, regardless of antihypertensive agent. However, the proportion of blood pressures in the severe range by each postpartum day was higher in the first ten days postpartum for those discharged on labetalol as compared to nifedipine (Figure 1). While the effect size was greater for postpartum systolic blood pressures (Supplemental Figure 1), the relationship persisted for the proportion of severe diastolic blood pressures (Supplemental Figure 2).

## Structured discussion/comment

### Principal findings

In our cohort, in line with prior studies, discharge on single-agent labetalol was associated with an approximately 2.5-fold odds of postpartum readmission when compared with discharge on single-agent nifedipine. We hypothesized that enrollment and participation in a remote monitoring program to allow for immediate assessment and management of severe postpartum hypertension may counteract any relationship between BP medication at discharge and unplanned care utilization in the postpartum period. Additionally, by including only those enrolled in a remote BP monitoring program, we were able to explore postpartum BP patterns and incidence of severe hypertension by type of discharge medication. Despite this, we found that those discharged on labetalol have a higher proportion of BPs in the severe range by each day postpartum in the first 10 days after delivery, which translated to greater odds of postpartum hospital readmission compared with those discharged on nifedipine.

### Results

Our findings contribute to the growing body of literature demonstrating an increased risk of postpartum hospital readmission when discharged on labetalol monotherapy. In a large single-center cohort study published in 2022 of 14,112 patients discharged on labetalol, 9,001 patients discharged on nifedipine, and 1,364 discharged on both agents, the rate of hospital readmission was significantly higher for those discharged on labetalol monotherapy (4.5% vs 2.7%, aOR 1.57, 95% CI 1.29–1.93).^10^ Likewise, a retrospective study of approximately 6,000 patients published in 2023 demonstrated a 65% decreased risk of hospital readmission for patients discharged on nifedipine monotherapy and 56% decreased risk of hospital admission for patients discharged on combination therapy.^11^ Similarly, a 2024 retrospective study found that discharge with a prescription for nifedipine alone or multiple antihypertensive medications was associated with a lower incidence of readmission, whereas prescription for labetalol alone was associated with an increased risk for readmission.^12^

Recently, Lovgren et al performed a randomized controlled trial of 323 postpartum patients assigned to receive nifedipine XL 30 milligrams twice daily vs labetalol 200 milligrams three times daily with a primary outcome of postpartum hospital readmission. This study found that the postpartum readmission rate was 88% lower in the nifedipine group as compared with the labetalol group (1.2% vs 8.1%, aOR 0.12).^20^

In utilizing the novel approach of TIS, we are able to better assess the relative amount of each medication a patient is prescribed at discharge, acknowledging the complexity of the dosing regimens for these agents. The TIS for nifedipine was significantly higher than for labetalol upon discharge, likely related to the dosing increments (i.e. the lowest dose of nifedipine is equal to a TIS of 0.25, while there are more flexible dosing options for labetalol). Given that the minimum TIS for labetalol is much lower than for nifedipine, it is possible that a relative underdosing is occurring for labetalol as compared to nifedipine, which could explain our and others’ findings. It is also possible that this is compounded by difficulties with adherence and frequent dosing for individuals prescribed labetalol. Further, our study utilized granular systolic and diastolic individual blood pressure data to assess blood control postpartum. Thus, while prior studies have used postpartum readmission as a surrogate for poor blood pressure control, our study was able to evaluate home postpartum blood pressure measures directly.

### Clinical implications

In the United States, postpartum hypertension is the leading cause of readmission following delivery hospitalization, comprising greater than 20% of readmissions within 30 days. At the institutional level, this serves as a quality improvement metric linked to provider reimbursement as well as a significant contributor to health care cost and utilization of resources.^2^ On a patient-facing level, there has been a critical shift in focus to the fourth trimester, acknowledging the critical nature of mother-infant bonding, habit-building, and engagement in long-term physical and mental health that occurs in the weeks following birth. This critical period may be irreparably fragmented by postpartum hospitalizations.

While the relationship between postpartum readmission and postpartum depression has not been definitively explored, preeclampsia has been established as a risk factor for postpartum depression.^21–25^ Presumably, a decreased risk of hospital readmission due to appropriate antihypertensive selection could yield improved critical bonding within the maternal-infant dyad, thus leading to enhanced postpartum mental health overall.

The concept that nifedipine is a more efficacious agent than labetalol for HDP in the postpartum period has biological plausibility. While the exact pathophysiology of postpartum preeclampsia is yet to be fully elucidated, there is a clear component of volume overload which contributes to significant elevations in blood pressure as fluid is re-mobilized to the intravascular space in the days following delivery.^26–29^ This mobilization of fluid into the intravascular space peaks during postpartum days 3–6, a period in which many women have already been discharged from the hospital.^28^ This pathophysiology is supported by the noted improvement in blood pressure control at 7 days postpartum for women with HDP who received a five-day course of oral furosemide.^29^ Further, in a study assessing brain natriuretic peptide (BNP) levels, a useful marker in diagnosing fluid overload, the median value for those with postpartum preeclampsia was noted to be 424 pg/mL (normal <100 pg/mL).^30^ While normative values for BNP in pregnancy and postpartum have not been clearly established, this nevertheless provides valuable insight into the role of volume overload in exacerbation of postpartum hypertension.

As compared to labetalol, nifedipine causes a significant increase in urinary flow rate combined with an increase in fractional excretion of sodium at the level of the proximal renal tubule, leading to an increased diuretic and natriuretic effect.^31–32^ In this manner, dual effects of decreased peripheral vascular resistance and improved diuresis can positively impact patients with HDP in the postpartum period well beyond their delivery hospitalization, and particularly during postpartum days 3-6 when intravascular fluid mobilization is at its highest. Conversely, labetalol decreases peripheral vascular resistance with minimal effect on overall volume status.^33^ These mechanistic differences may underscore the clinical findings of decreased readmission rates for those discharged on nifedipine postpartum.

### Research implications

As previously noted, published literature regarding readmission rates in the postpartum period following HDP have consistently noted an increased rate of readmission with discharge on labetalol monotherapy. This has remained true across multiple studies with diverse patient populations throughout several geographic regions. While these data are compelling, all published studies have either been retrospective in nature or single-center. Future study calls for a large, multi-center randomized controlled trial to corroborate these findings. Further, a meta-analysis could inform the effect size across the diverse cohorts that have been studied to-date to increase external validity.

### Strengths and limitations

Strengths of our study include the granularity of blood pressure data allowed for through our remote home BP monitoring database. Accordingly, systolic and diastolic blood pressures were monitored throughout an individual’s outpatient course, allowing for finite examination of the degree of postpartum blood pressure elevation beyond an individual’s delivery admission. This is particularly important during postpartum days 3–6 when intravascular fluid shifts are highest. Thus, while prior studies have used postpartum readmission as a surrogate for poor blood pressure control, our study is able to evaluate outpatient postpartum blood pressure control directly using home BP data. Additionally, our study employs a diverse patient population and broad inclusion criteria, increasing its external validity.

However, our study has several limitations. Primarily, the study is retrospective in nature and lacks the ability to control for confounding factors contributing to readmission. While a multivariate regression was performed, there remains the possibility that unidentified confounders contributed to differential rates of readmission. As the study is retrospective, it is possible that other practice pattern changes or temporal trends occurred during the study period that remain unaccounted for regarding readmission. There are no standard institutional criteria for hypertension-related readmissions, and thus provider variation may have led to differences in the primary outcome. Additionally, medication dosages were confined to the time of discharge; the degree to which medication agents and/or dosages were adjusted following discharge and at subsequent postpartum visits was not captured within our dataset. Data regarding number of contacts within our remote monitoring program are not available. Further, medication adherence data are not available. Lastly, as this was a single-center study, it is possible that readmissions and/or ED visits outside our hospital system were not captured.

## Conclusions

In this study of postpartum health care utilization following HDP with discharge on single-agent labetalol or nifedipine, discharge on single-agent labetalol was associated with 2.5-fold increased odds of postpartum readmission compared to discharge on single-agent nifedipine. The etiology for these findings is suspected to be multifactorial. Nifedipine and labetalol have differing mechanisms of action, with a diuretic component in nifedipine that is not present in labetalol. Given profound intravascular volume shifts postpartum, this diuretic effect may be crucial when managing severe postpartum hypertension. Additionally, it is possible that differing dosing strategies may lead to differential medication adherence and relative underdosing of labetalol compared to nifedipine. Our findings add to the growing body of literature supporting an increase in readmission with discharge on labetalol monotherapy compared with nifedipine and raise questions about the most appropriate first-line antihypertensive medication for individuals with HDP.

## Supplementary Material

Supplemental figures

Supplemental tables

Supplementary material associated with this article can be found in the online version at doi:10.1016/j.ajogmf.2025.101836.

## Figures and Tables

**TABLE 1:** Demographic and clinical characteristics by antihypertensive agent at discharge

	Nifedipine XL (N=1019)	Labetalol (N=488)	*P*-value
Age (yrs)	30.8±6	30.9±6.1	.69
Primiparous (%)	497 (48.8%)	256 (52.5%)	.18
Gestational age at delivery (wks)	36.4±3.7	36.5±3.3	.76
Early pregnancy BMI (kg/m2)	28 [23.3, 33.8]	29.5 [24.7, 36.8]	<.001
Self-identified race			<.001
Caucasian	649 (63.7%)	381 (78.0%)	
Black	305 (29.9%)	83 (17.0%)	
Asian	29 (2.9%)	10 (2.1%)	
Other	36 (3.5%)	14 (2.9%)	
Insurance status			.17
Commercial	534 (52.4%)	278 (57.0%)	
Medicaid/Medicare	467 (45.8%)	205 (42.0%)	
Self-Pay/Other	18 (1.8%)	5 (1.0%)	
Gestational diabetes (%)	111 (10.9%)	62 (12.7%)	.30
Chronic hypertension (%)	192 (18.8%)	166 (34.0%)	<.001
Inpatient magnesium use (%)	661 (64.9%)	286 (58.6%)	.02
Mode of delivery (%)			.13
Spontaneous vaginal delivery	512 (50.2%)	225 (46.1%)	
Cesarean delivery	507 (49.8%)	263 (53.9%)	
Therapeutic intensity score (TIS)	0.25 [0.25–0.50]	0.25 [0.17–0.33]	<.001

Data presented as Mean±SD, Median [IQR], N (column %).

**TABLE 2:** Health care utilization by antihypertensive agent at discharge

	Nifedipine XL (N=1019)	Labetalol (N=488)	*P*-value
Emergency room visit postpartum	106 (10.4%)	56 (11.5%)	.53
	Ref	OR 1.06 (0.72–1.57)	.53
	Ref	aOR 1.01 (0.67–1.52)	.95
Hospital readmission postpartum	40 (3.9%)	45 (9.2%)	<.001
	Ref	OR 2.49 (1.60–3.86)	<.001
	Ref	aOR 2.70 (1.57–4.65)	<.001

OR, oddsratio.

aOR: adjusted odds ratio, adjusted for severity of hypertensive disorder, therapeutic intensity score, race, and BMI

**TABLE 3:** Postpartum blood pressures by antihypertensive agent at discharge

	Nifedipine XL (N=1019)	Labetalol (N=488)	*P*-value
Severe hypertension after discharge (BP≥160/110 mmHg)	182 (18%)	106 (22%)	.07
Mean systolic home BP (mmHg)	126±9	127±9	.32
Mean diastolic home BP (mmHg)	83±6	82±6	<.001
Maximum systolic home BP (mmHg)	147±13	150±15	.004
Maximum diastolic home BP (mmHg)	98±10	96±10	.005

Statistics presented as Mean±SD, N (column %).

## AJOG MFM at a Glance

### Why was this study conducted?

The objective was to compare postpartum health care utilization following hypertensive disorders of pregnancy for individuals discharged on single-agent labetalol compared to single-agent nifedipine.

### Key findings

While emergency room utilization was similar following discharge, postpartum readmission was significantly higher in individuals discharged on single-agent labetalol. Additionally, those discharged on labetalol had a higher proportion of blood pressures in the severe range by each day postpartum during the immediate postpartum period compared to those discharged on nifedipine.

### What does this add to what is known?

The finding of increased postpartum readmission following hypertensive disorder of pregnancy after discharge on single-agent labetalol as compared to single-agent nifedipine persists in a model that utilizes postpartum home blood pressure monitoring as standard of care.
