# Supplementary material for: Postpartum health-care utilization and blood pressure control by antihypertensive agent in hypertensive disorders of pregnancy
Source: Am J Obstet Gynecol MFM. Author manuscript; Available in PMC 2026 Jun 22. (PMC13285104; doi:10.1016/j.ajogmf.2025.101836)
Supplement: Supplemental figures [file NIHMS2182595-supplement-Supplemental_figures.docx]

**Supplemental Figure 1**: Proportion of Systolic Blood Pressures in the Severe Range by Antihypertensive Agent at Discharge

**Supplemental Figure 2**: Proportion of Diastolic Blood Pressures in the Severe Range by Antihypertensive Agent at Discharge
