# Supplementary material for: Postpartum health-care utilization and blood pressure control by antihypertensive agent in hypertensive disorders of pregnancy
Source: Am J Obstet Gynecol MFM. Author manuscript; Available in PMC 2026 Jun 22. (PMC13285104; doi:10.1016/j.ajogmf.2025.101836)
Supplement: Supplemental tables [file NIHMS2182595-supplement-Supplemental_tables.docx]

**Supplemental Table 1. Hypertension-Related Health Care Utilization by Antihypertensive Agent at Discharge**

|  | **Nifedipine XL (N=1019)** | **Labetalol (N=488)** | **p-value** |
| --- | --- | --- | --- |
| Hypertension-related emergency room visit postpartum | 71 (7.0%) | 42 (8.6%) | 0.26 |
|  | Ref | OR 1.26 (0.84-1.87) | 0.26 |
|  | Ref | aOR 1.26 (0.81-2.00) | 0.95 |
| Hypertension-related  hospital readmission postpartum | 34 (3.3%) | 41 (8.4%) | <0.001 |
|  | Ref | OR 2.66 (1.66-4.24) | <0.001 |
|  | Ref | aOR 2.76 (1.62-4.71) | <0.001 |

OR: odds ratio

aOR: adjusted odds ratio, adjusted for severity of hypertensive disorder, therapeutic intensity score, race, and BMI

**Supplemental Table 2. Health Care Utilization by Antihypertensive Agent at Discharge in Individuals with Chronic Hypertension**

|  | | |  |
| --- | --- | --- | --- |
|  | **Nifedipine XL (N=192)** | **Labetalol (N=166)** | **p-value** |
| Emergency room visit postpartum | 22 (11.5%) | 18 (10.8%) | 0.85 |
|  | Ref | OR 0.94 (0.49-1.82) | 0.85 |
|  | Ref | aOR 0.85 (0.37-1.96) | 0.70 |
| Hospital readmission postpartum | 13 (6.8%) | 19 (11.5%) | 0.13 |
|  | Ref | OR 1.78 (0.85-3.72) | 0.13 |
|  | Ref | aOR 2.08 (0.84-5.15) | 0.12 |

OR: odds ratio

aOR: adjusted odds ratio, adjusted for severity of hypertensive disorder, therapeutic intensity score, race, and BMI

**Supplemental Table 3. Health Care Utilization by Antihypertensive Agent at Discharge in Individuals without Chronic Hypertension**

|  | |  |  |
| --- | --- | --- | --- |
|  | **Nifedipine XL (N=827)** | **Labetalol (N=322)** | **p-value** |
| Emergency room visit postpartum | 84 (10.2%) | 38 (11.8%) | 0.42 |
|  | Ref | OR 1.18 (0.79-1.78) | 0.42 |
|  | Ref | aOR 1.11 (0.69-1.80) | 0.66 |
| Hospital readmission postpartum | 27 (3.3%) | 26 (8.1%) | <0.001 |
|  | Ref | OR 2.6 (1.49-4.53) | <0.001 |
|  | Ref | aOR 2.88 (1.43-5.80) | 0.003 |

OR: odds ratio

aOR: adjusted odds ratio, adjusted for severity of hypertensive disorder, therapeutic intensity score, race, and BMI
